# Melioidosis on Phu Quoc Island, Vietnam: a retrospective case series from a remote tropical setting

**DOI:** 10.1186/s12879-026-14150-4

**Published:** 2026-08-13

**Authors:** Duy-Cuong Le, Dinh-Dung Nguyen, Thuy-Hang Thi Nguyen, Hanh Vu, Xuan-Dat Mai, Thu-Ha Nguyen, Nu Thi Nguyen, Huyen-Trang Thi Le, Thao-Loan Thi Truong, Hong-Sa Thi Nguyen, Mai Thi Nguyen, Nhat-Tan Nguyen Hoang, Linh Thi Nguyen, Anh-Khoi Nguyen, Ha Hong Pham, Thien-Thanh Le, Thanh-Ha Thi Ly, Xuan-Hung Nguyen, Ngoan Tran Le, Hoa Thi Thanh Hoang, Thu-Hien Thi Tran, Quoc-Hung Doan, Duc-Dinh Vu

**Affiliations:** 1 Vinmec Phu Quoc, Vinmec International Hospital, Phu Quoc Special Zone, An Giang, Vietnam; 2 Department of Laboratory Medicine, Vinmec Riverside, Vinmec International Hospital, Hanoi, 100000 Vietnam; 3 Vinmec - VinUni Institute of Immunology, College of Health Sciences, VinUniversity, Hanoi, 100000 Vietnam; 4 Vinmec Times City, Vinmec International Hospital, Hanoi, 100000 Vietnam; 5 Innovations in Health Sciences, VinUniversity, Hanoi, Vietnam; 6 Scientific Research Management Center, Vinmec International Hospital, Hanoi, 100000 Vietnam; 7 Vinmec Ocean Park 2, Vinmec International Hospital, Hung Yen, Vietnam

**Keywords:** Melioidosis, Whitmore disease, *Burkholderia pseudomallei*, sepsis, septic shock, Phylogenetic analysis

## Abstract

**Background:**

Melioidosis, caused by *Burkholderia pseudomallei*, is an important but frequently under-recognized cause of severe infection in tropical regions, including Vietnam. Although cases have been reported from several parts of the country, published clinical data from remote island settings remain scarce. To our knowledge, no clinical case series of melioidosis from Phu Quoc Island has been previously reported. This study describes the clinical presentations, underlying comorbidities, management, and outcomes of patients diagnosed with melioidosis at a tertiary hospital on Phu Quoc Island, Vietnam.

**Case presentation:**

We retrospectively reviewed all patients with culture-confirmed melioidosis admitted to Vinmec Phu Quoc General Hospital between July 2022 and February 2026. Archived isolates underwent molecular confirmation using a TTS1-targeted quantitative polymerase chain reaction (qPCR) assay, followed by recA gene sequencing. Nine patients were included. Clinical, laboratory, microbiological, radiological, treatment, and outcome data were extracted from medical records.

Pneumonia was the predominant manifestation, occurring in seven patients, and eight patients presented with bacteremic disease. Diabetes mellitus was the most common underlying condition (7/9), followed by chronic liver disease and previous tuberculosis. Most patients were admitted with sepsis, including four cases complicated by septic shock. Two patients developed rapidly progressive pulmonary melioidosis with acute respiratory distress syndrome (ARDS) and multiple organ dysfunction syndrome (MODS) and died despite intensive antimicrobial and supportive therapy, including one previously healthy individual. The remaining seven patients survived. TTS1 qPCR confirmed all available isolates, while recA sequencing demonstrated the presence of at least two closely related sequence variants among circulating isolates. Antimicrobial susceptibility testing by broth microdilution showed preserved susceptibility to amoxicillin/clavulanic acid, ceftazidime, imipenem, and trimethoprim/sulfamethoxazole in all tested isolates.

**Conclusion:**

This report represents, to our knowledge, the first published clinical case series of melioidosis from Phu Quoc Island. The findings highlight the broad clinical spectrum of melioidosis and its potential for rapid progression to life-threatening pneumonia, septic shock, and multiorgan failure. Clinicians should maintain a high index of suspicion in patients presenting with severe pneumonia or sepsis, particularly during the rainy season and among individuals with diabetes mellitus or chronic liver disease. Strengthening conventional microbiological capacity, including timely blood culture, accurate organism identification, and standardized antimicrobial susceptibility testing, remains essential for improving diagnosis and patient outcomes in geographically isolated settings.

**Supplementary information:**

The online version contains supplementary material available at https://doi.org/10.1186/s12879-026-14150-4.

## Introduction

Melioidosis is a life-threatening infectious disease caused by the environmental Gram-negative bacillus *Burkholderia pseudomallei*. It is primarily endemic to tropical and subtropical regions, with the highest burden reported in Southeast Asia and northern Australia. Increasing case reports from South Asia, China, and parts of South America and Africa indicate a broader global distribution than previously recognized [1, 2]. Globally, melioidosis is estimated to cause up to 165,000 cases and 89,000 deaths annually [1].

*B. pseudomallei* resides in soil and surface water, thriving particularly in wet environments during the rainy season. Infection occurs primarily via percutaneous inoculation, inhalation, or ingestion of contaminated soil or water. Occupational and environmental exposures - especially among rice farmers, agricultural laborers, and construction workers - significantly increase the risk [3, 4].

Clinically, melioidosis is often referred to as “the great mimicker” because of its wide spectrum of manifestations, ranging from localized skin and soft tissue infections to pneumonia, fulminant sepsis, and disseminated multi-organ disease. Pneumonia and bacteremia are the most common clinical presentations, although neurologic involvement, genitourinary disease, and deep visceral abscesses have also been described [5]. Severe disease occurs more frequently in individuals with underlying comorbidities, particularly diabetes mellitus, chronic kidney disease, and hazardous alcohol use [1]. In developed countries, an increasing number of cases are related to international travel and importation. No licensed vaccine is currently available. Strengthening microbiological laboratory capacity often leads to the detection of *B. pseudomallei* in new regions, with recent reports from India, southern China, Brazil, and Malawi [1]. Given diagnostic limitations in many low- and middle-income countries, the true geographic range is likely broader, with undetected endemicity in multiple tropical settings.

Melioidosis is associated with substantial mortality in several parts of Southeast Asia, even where hospital-based care is available. Case fatality rates of up to 80% have been reported in rural Cambodia among patients who did not receive timely appropriate antimicrobial therapy. Mortality remains high in northeastern Thailand (approximately 40%) and Malaysia (33–54% overall, and up to 65% in patients with septic shock) [6–8]. Diagnosis is challenging because clinical features are non-specific and may mimic tuberculosis, community-acquired pneumonia, or sepsis of unknown origin. Definitive diagnosis relies on culture and accurate identification of *B. pseudomallei*, but access to microbiological diagnostic capacity is often limited in remote or resource-constrained settings [9, 10].

Treatment of melioidosis consists of a two-phase regimen, including an initial intensive intravenous phase followed by a prolonged oral eradication phase to prevent relapse [11]. Studies from northeastern Thailand have demonstrated that relapse risk is influenced by the choice and duration of eradication therapy, with higher recurrence rates observed following shorter courses of oral treatment [12]. More recent data indicate that recurrence rates have declined over time, likely reflecting improvements in antimicrobial regimens, clinical management, and treatment adherence [7].

Despite the recognized burden of melioidosis in Southeast Asia, published clinical data from Vietnam remain limited, consisting mainly of sporadic case reports and small series from selected regions. To our knowledge, no clinical case series of melioidosis from Phu Quoc Island has been previously published. In this study, we report nine patients with culture-confirmed melioidosis diagnosed at the only tertiary care hospital on Phu Quoc Island. We aim to describe their clinical presentations, underlying comorbidities, management, and outcomes, and to highlight diagnostic and clinical challenges relevant to remote island and resource-limited healthcare settings.

## Methods

### Study design

This retrospective case series was conducted at Vinmec Phu Quoc General Hospital, where culture-based identification of *Burkholderia pseudomallei* was routinely performed during the study period. Patients with culture-confirmed melioidosis admitted between July 2022 and February 2026 were retrospectively identified from microbiology laboratory records. Archived bacterial isolates were subsequently subjected to molecular confirmation by qPCR targeting the type III secretion system 1 (TTS1) gene. Only cases with qPCR-confirmed *B. pseudomallei* infection were included in the final analysis.

### Study population

Patients of all ages and sexes with qPCR-confirmed melioidosis were eligible for inclusion. Clinical diagnosis of melioidosis was established based on compatible clinical features and microbiological confirmation. Demographic characteristics, underlying comorbidities, clinical presentations, laboratory findings, antimicrobial treatment, and clinical outcomes were retrieved from electronic medical records using a standardized data collection approach.

### Microbiological methods

Clinical specimens, including blood and site-specific samples when available, were processed according to standard microbiological procedures routinely used in the hospital laboratory. Blood cultures were incubated using automated blood culture systems, and subcultures were performed on blood agar, MacConkey agar, and brain heart infusion broth. Bacterial identification was initially performed using the VITEK 2 automated system (bioMérieux, USA). Identification of non-fermenting Gram-negative bacilli was further confirmed using the API 20 NE system (bioMérieux, USA), according to the manufacturer’s instructions.

Antimicrobial susceptibility testing (AST) was performed using the broth microdilution method in accordance with the Clinical and Laboratory Standards Institute (CLSI) recommendations for *B. pseudomallei*. Minimum inhibitory concentrations (MICs) were determined using commercially prepared Sensititre microdilution plates (THAN1F; Thermo Fisher Scientific, Cleveland, OH, USA) with Mueller–Hinton broth (T3462; Thermo Fisher Scientific, Cleveland, OH, USA) according to the manufacturer’s instructions. Briefly, bacterial suspensions prepared from freshly cultured isolates were inoculated into the microdilution plates and incubated under aerobic conditions. MIC endpoints were determined using a Sensititre Manual Viewbox (Thermo Fisher Scientific, Cleveland, OH, USA). The antimicrobial agents evaluated in the panel included amoxicillin–clavulanate (AMC), ceftazidime (CAZ), imipenem (IMP), and trimethoprim–sulfamethoxazole (SXT). MIC values were interpreted according to CLSI breakpoints for *B. pseudomallei* as described in CLSI document M45, Methods for Antimicrobial Dilution and Disk Susceptibility Testing of Infrequently Isolated or Fastidious Bacteria [13]. Tetracycline, although included among the antimicrobial agents for which CLSI M45 provides interpretive criteria for *B. pseudomallei*, was not available in the commercial microdilution panel used in this study.

### Molecular characterization

DNA extraction: Genomic DNA (gDNA) was extracted from individual bacterial colonies. Briefly, bacterial cells were mechanically disrupted using a TissueLyser II system (QIAGEN, Hilden, Germany) at 20 Hz for 4 min. DNA extraction was subsequently performed using the QIAamp PowerFecal Pro DNA Kit (QIAGEN, Hilden, Germany) according to the manufacturer’s instructions. DNA was eluted in 50 μL of nuclease-free water and quantified using a NanoDrop spectrophotometer (Thermo Fisher Scientific, Waltham, MA, USA). Extracted DNA was subsequently used for quantitative real-time PCR (qPCR) and Sanger sequencing.

Real-time PCR confirmation of *Burkholderia pseudomallei*: Species identification was confirmed by qPCR targeting the type III secretion system 1 (TTS1) gene, a highly specific marker for *B. pseudomallei*. Primers and hydrolysis probe were adopted from Novak et al. (2006) [14]. The forward primer sequence was 5′-CGTCTCTATACTGTCGAGCAATCG-3′ and the reverse primer sequence was 5′-CGTGCACACCGGTCAGTATC-3′, generating an expected amplicon of 115 bp. The probe sequence was 5′-CCGGAATCTGGATCACCACCACTTTCC-3′, labeled with 6-carboxyfluorescein (FAM) at the 5′ end and Black Hole Quencher-1 (BHQ-1) at the 3′ end.

Each 10 μL reaction mixture contained 0.4 μL of template DNA, 1× eQo 1-Step ToughMix, 500 nM of each primer, and 100 nM probe. Amplification and fluorescence detection were performed on an Applied Biosystems 7500 Real-Time PCR System (Applied Biosystems, Foster City, CA, USA) under the following cycling conditions: initial denaturation at 95 °C for 2 min, followed by 40 cycles of 95 °C for 10 s and 60 °C for 45 s. A no-template control (nuclease-free water) and a non-target bacterial DNA control consisting of macrolide-resistant Mycoplasma pneumoniae genomic DNA were included in each run to monitor contamination and non-specific amplification.

recA gene amplification and Sanger sequencing: Phylogenetic analysis based on the recA gene was performed to assess the genetic relatedness of the study isolates and their relationship to representative reference strains. Primers were used to amplify an approximately 869-bp fragment. The forward primer sequence was 5′-GATCGARAAGCAGTTCGGCAA-3′ and the reverse primer sequence was 5′-TTGTCCTTGCCCTGRCCGAT-3′ [15].

PCR amplification was carried out in a 12.5 μL reaction mixture containing 0.5 μL of template DNA, 1× GoTaq Master Mix (Promega, Madison, WI, USA), and 400 nM of each primer. Amplification products were verified by agarose gel electrophoresis and purified using AMPure XP beads (Beckman Coulter Diagnostics, Brea, CA, USA). Purified PCR products were quantified using a NanoDrop spectrophotometer prior to sequencing.

Cycle sequencing reactions were performed using the BigDye Terminator v3.1 Cycle Sequencing Kit (Applied Biosystems, Foster City, CA, USA) in a final volume of 10 μL containing 0.5 μL of BigDye Terminator reagent, 1× sequencing buffer, and approximately 20 ng of purified PCR product. Cycling conditions consisted of an initial denaturation at 96 °C for 2 min, followed by 25 cycles of 96 °C for 10 s, 50 °C for 10 s, and 60 °C for 2 min. Sequencing products were purified using BigDye XTerminator Purification Kit (Applied Biosystems) according to the manufacturer’s instructions and analyzed using an Applied Biosystems 3500 Dx Genetic Analyzer.

Sequence and phylogenetic analysis: Consensus sequences were compared against the National Center for Biotechnology Information (NCBI) database using the Basic Local Alignment Search Tool (BLAST) to confirm species identity. Sequences obtained from the study isolates and selected reference strains were aligned using CLUSTAL W [16]. The list of reference strains included in the phylogenetic analysis is provided in Supplementary Table S1. Phylogenetic relationships were inferred using the maximum-likelihood method implemented in IQ-TREE, and the resulting phylogenetic tree was visualized using Interactive Tree of Life (iTOL) version 7.

### Data collection

Clinical, microbiological, and laboratory data were extracted from electronic medical records using a standardized data collection form. Collected variables included demographic characteristics, epidemiological exposure, underlying comorbidities, clinical manifestations, laboratory findings, antimicrobial therapy, and clinical outcomes. Disease severity was assessed retrospectively using the Sequential Organ Failure Assessment (SOFA) score. SOFA scores were calculated according to the original scoring system using the worst clinical and laboratory parameters recorded within the first 24 hours of hospital admission. All extracted data were anonymized prior to analysis.

### Statistical analysis

Descriptive statistics were used to summarize patient characteristics, clinical features, laboratory findings, treatment, and outcomes. Continuous variables are presented as medians with interquartile ranges (IQR), whereas categorical variables are presented as counts and percentages. For paired comparisons between hospital admission and the eradication phase, only patients who survived and completed follow-up assessments at both time points were included (*n* = 7). Due to the small sample size and non-normal distribution of the data, the two-tailed Wilcoxon signed-rank test was used for paired analyses. Statistical analyses were performed using GraphPad Prism version 10.3.1 (GraphPad Software, San Diego, CA, USA). A two-sided *p*-value < 0.05 was considered statistically significant.

## Results

### Characteristics of the patients and epidemiological data

**Fig. 1:**
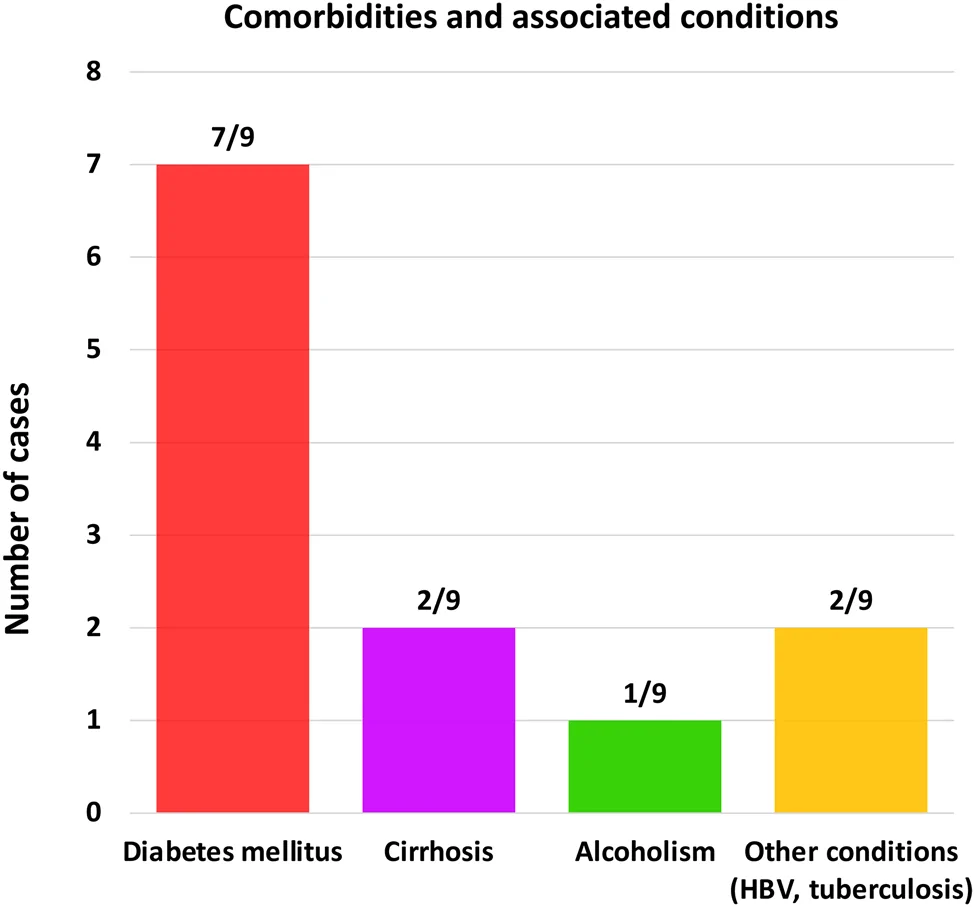
Distribution of comorbidities and associated conditions among patients with melioidosis (*n* = 9). Diabetes mellitus was the most frequent comorbidity, present in 7 of 9 patients. Other conditions included hepatitis B virus infection, or tuberculosis (2/9), cirrhosis (2/9), and hazardous alcohol use (1/9)

Among nine patients with qPCR-confirmed melioidosis treated between July 2022 and February 2026, the median age was 53 years (range, 34–77), and six patients were male (66.7%). Diabetes mellitus was the most common comorbidity, present in seven patients (77.8%), followed by cirrhosis (2/9). All patients were permanent residents of Phu Quoc Island and reported no recent travel to the mainland or other known endemic regions. Notably, seven of nine cases occurred during the rainy season (May to October). The interval from symptom onset to hospital admission ranged from 1 to 10 days (Table 1, Fig. 1).

**Table 1:** Demographic characteristics and epidemiological features of nine patients with culture-confirmed melioidosis

Case	Age (Years)	Sex	Residence	Admission date	Interval from symptom onset to admission (days)	Comorbidities / risk factors	Possible portal of entry
1	51	F	Phu Quoc Island	Jul 2022	1	Diabetes mellitus	Not identified
2	38	M	Phu Quoc Island	Aug 2022	4	Diabetes mellitus	Not identified
3	35	M	Phu Quoc Island	May 2023	5	Diabetes mellitus	Not identified
4	56	M	Phu Quoc Island	Aug 2023	10	Chronic hepatitis B; Child–Pugh C cirrhosis with hepatocellular carcinoma and ascites	Not identified
5	34	F	Phu Quoc Island	Jul 2024	3	None	Purulent tonsillitis (suspected)
6	77	F	Phu Quoc Island	Feb 2026	3	Diabetes mellitus	Oropharyngeal route (suspected)
7	56	M	Phu Quoc Island	Sep 2024	3	Diabetes mellitus; tuberculosis	Not identified
8	53	M	Phu Quoc Island	Jan 2025	7	Alcohol-related cirrhosis; diabetes mellitus	Intramuscular injection (right thigh cellulitis)
9	57	M	Phu Quoc Island	May 2025	7	Diabetes	Acute purulent pharyngitis (suspected)

Among the nine cases, no clear environmental exposure source could be identified in five patients (Cases 1–4 and 7), although all had underlying conditions - most commonly diabetes mellitus or cirrhosis - that may predispose to melioidosis. In the remaining four cases, potential portals of entry were identified. In three patients (Cases 5, 6, and 9), upper respiratory tract symptoms preceded the onset of melioidosis requiring hospital admission. Case 5 developed symptoms following purulent tonsillitis, Case 6 initially presented with fever, rhinorrhea, and cough, and Case 9 developed symptoms following acute purulent pharyngitis. Although a definitive route of acquisition could not be established, these preceding upper respiratory tract manifestations raise the possibility of respiratory or oropharyngeal acquisition. Case 8, who had alcohol-related cirrhosis and diabetes mellitus, resided in a rural area of Phu Quoc Island characterized by natural streams and seasonally waterlogged soil, environments known to harbor *B. pseudomallei*. The patient presented with rapidly progressive cellulitis of the right thigh and reported repeated intramuscular injections at a private clinic before symptom onset. Although the environmental source of infection could not be determined, inoculation through the injection site was considered a possible route of entry.

Overall, in more than half of the cases (5/9), the exact source of infection could not be determined. In the remaining patients, preceding upper respiratory tract manifestations - including purulent tonsillitis, acute purulent pharyngitis, rhinorrhea, and cough - or a history of repeated intramuscular injections were considered potential routes of acquisition. However, the environmental source of infection could not be determined in any individual case. (Table 1, Fig. 1).

### Clinical manifestations

Clinical manifestations and outcomes are summarized in Table 2.

**Table 2:** Clinical presentation, severity, management, and outcomes of patients with melioidosis

Case	Primary clinical presentation	SOFA score at admission	ICU/HDU admission	Mechanical ventilation	Vasopressor support	Renal replacement therapy	Initial antimicrobial therapy	Outcome
1	Pneumonia with sepsis	3	ICU	No	No	No	Ceftriaxone	Survived; referred to tertiary hospital
2	Pneumonia with sepsis and intracerebral hemorrhage	7	ICU	Yes	No	No	Meropenem + Levofloxacin	Survived; referred to tertiary hospital
3	Meningoencephalitis, diabetic ketoacidosis, and sepsis	3	HDU	Yes	No	No	Ceftriaxone	Survived; referred to tertiary hospital
4	Spontaneous bacterial peritonitis with sepsis	10	No	No	No	No	Meropenem	Fully recovered
5	Pneumonia with septic shock, ARDS, and MODS	12	ICU	Yes	Yes	No	Meropenem + Amikacin + Vancomycin	Deceased
6	Severe bilateral pneumonia with septic shock, ARDS, and MODS	11	ICU	No*	Yes	No	Meropenem + Levofloxacin + Amikacin	Deceased
7	Pneumonia with septic shock	11	ICU	Yes	Yes	CVVHDF	Meropenem + Amikacin + Vancomycin	Fully recovered
8	Cellulitis, pneumonia, and sepsis	5	No	No	No	No	Meropenem + Vancomycin	Survived; referred to tertiary hospital
9	Pneumonia with septic shock, DIC, and MODS	14	ICU	Yes	Yes	No	Meropenem + Vancomycin	Fully recovered

*Mechanical ventilation was indicated because of severe ARDS, but endotracheal intubation was declined by the family; CVVHDF - Continuous veno-venous hemodiafiltration; HDU-High-Dependency Unit; ICU-Intensive care unit

All nine patients presented with invasive melioidosis requiring hospital admission. Most patients had clinical features consistent with sepsis, accompanied by markedly elevated inflammatory markers, including procalcitonin (PCT), C-reactive protein (CRP), and white blood cell count (WBC). Septic shock developed in five patients (Cases 4, 5, 6, 7, and 9), all of whom had a Sequential Organ Failure Assessment (SOFA) score ≥ 10 at presentation.

Pneumonia was the predominant clinical manifestation, occurring in six of nine patients (Cases 1, 2, 5, 6, 7, 8, and 9). Notably, Cases 5 and 6 exhibited rapidly progressive pulmonary disease, with initially limited radiographic abnormalities that evolved within days into diffuse bilateral pulmonary infiltrates and severe respiratory failure. Both patients subsequently developed acute respiratory distress syndrome (ARDS), septic shock, and multiple organ dysfunction syndrome (MODS). Other clinical presentations included meningoencephalitis with diabetic ketoacidosis (Case 3), spontaneous bacterial peritonitis in a patient with advanced cirrhosis (Case 4), and severe cellulitis associated with pneumonia and sepsis (Case 8). Overall, seven patients required ICU or HDU admission, five underwent invasive mechanical ventilation, four required vasopressor support, and one received continuous veno-venous hemodiafiltration (CVVHDF). Despite intensive antimicrobial and supportive therapy, both patients with rapidly progressive pulmonary melioidosis (Cases 5 and 6) died, whereas the remaining seven patients survived (Table 2).

**Fig. 2:**
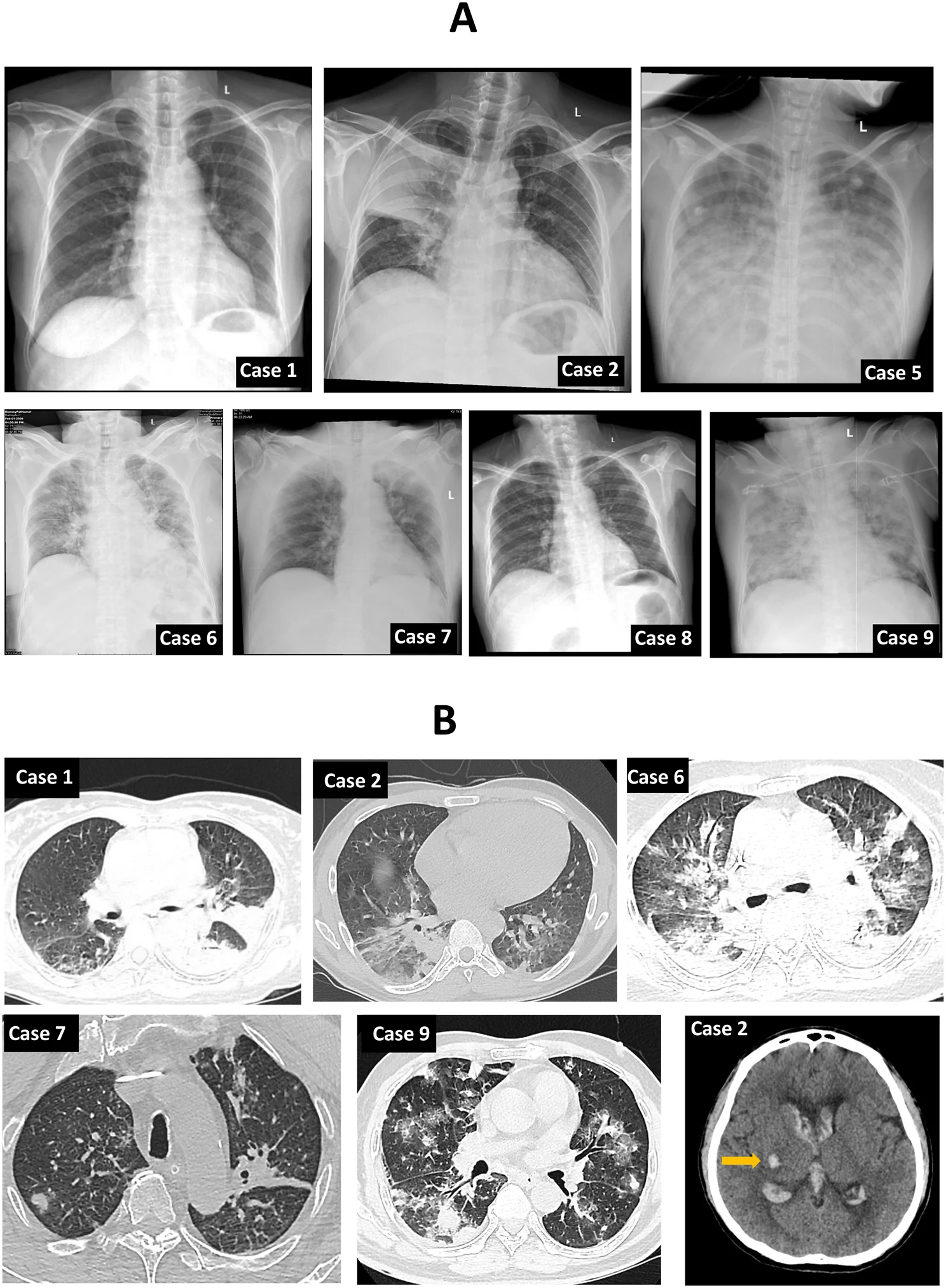
Radiological findings in patients with Melioidosis. Panel **A.** Chest radiographs of six patients with pulmonary melioidosis (cases 1, 2, 5, 6, 7, 8, and 9), demonstrating diverse pulmonary manifestations including pleural effusion, focal or multilobar consolidation, diffuse interstitial infiltrates, nodular opacities, and rapidly progressive bilateral air-space disease. Panel **B.** High-resolution computed tomography (HRCT) images from five patients (cases 1, 2, 6, 7, and 9), showing lobar consolidation, multifocal nodular infiltrates, cavitary lesions, diffuse ground-glass opacities, and air bronchograms, consistent with pulmonary melioidosis. Axial brain computed tomography image of Case 2 demonstrating a deep right hemispheric intracerebral hemorrhage (orange arrow) with intraventricular extension and surrounding cerebral edema

Among the seven patients with pulmonary melioidosis (Cases 1, 2, 5, 6, 7, 8, and 9), chest radiography demonstrated diverse pulmonary abnormalities (Fig. 2A). Common findings included diffuse interstitial opacities, patchy or confluent air-space consolidations, and pleural effusions. Radiographic manifestations ranged from focal lobar involvement (Case 2) to bilateral diffuse infiltrates (Cases 5, 6, and 7). Pleural effusion was observed in Cases 1 and 5, whereas Case 9 presented with multiple bilateral nodular and patchy pulmonary opacities. In Case 8, chest radiography demonstrated only mild bilateral peribronchovascular interstitial thickening despite systemic infection.

Chest CT was performed in five patients (Cases 1, 2, 6, 7, and 9) and revealed a broader spectrum of pulmonary abnormalities (Fig. 2B). Findings included lobar or segmental consolidation with air bronchograms (Cases 1 and 2), diffuse ground-glass opacities and bilateral alveolar consolidations (Case 6), multiple bilateral nodules with cavitary transformation (Case 7), and multifocal nodular consolidations that subsequently evolved into cavitary lesions during treatment (Case 9). Several patients demonstrated a combination of consolidation, ground-glass attenuation, and interstitial thickening, reflecting the heterogeneous radiological presentation of pulmonary melioidosis.

Notably, Cases 5 and 6 showed rapidly progressive pulmonary involvement. Initial imaging demonstrated relatively limited abnormalities, but within days both patients developed extensive bilateral pulmonary infiltrates with diffuse alveolar consolidation, resulting in ARDS and fatal outcomes. In contrast, Cases 7 and 9 exhibited gradual radiological improvement following appropriate antimicrobial therapy, although residual nodular and cavitary lesions persisted during follow-up imaging.

In addition to pulmonary findings, Case 2 initially demonstrated clinical improvement following antimicrobial therapy, with successful weaning from invasive mechanical ventilation to nasal cannula oxygen support after three days of treatment. However, on hospital day 4, the patient developed sudden left-sided hemiplegia. Brain computed tomography revealed a deep right hemispheric intracerebral hemorrhage with intraventricular extension and surrounding cerebral edema (Fig. 2B).

### Laboratory characteristics at admission and eradication phase

At hospital admission, most patients demonstrated marked hematological and biochemical abnormalities consistent with severe systemic infection (Table 3). Leukocytosis was observed in six of nine patients, with white blood cell (WBC) counts reaching 27.5 G/L in Case 4. In contrast, two patients (Cases 5 and 7) presented with profound leukopenia (WBC 2.5 and 2.9 G/L, respectively), a finding that may occur in severe sepsis. Neutrophilia was common, with neutrophil proportions exceeding 85% in six patients.

**Table 3:** Laboratory findings of patients at admission and eradication phases

Case/ Gender	Time point	Microbiological findings	WBC 4–10 (G/L)	NE 42–76(%)	PLT 150–450 (G/L)	PCT <0.046 (ng/ml)	Total bilirubin 5–21 (μmol/L)	Creatinine (μmol/L) ♂: 72–127 ♀: 58–96	D-Dimer ≤500 (ng/ml)
1/♀	**Admission**	*B. Pseudomallei* *Blood (2 sites)*	**15.9**	**88.3**	**151**	**1.68**	**18.3**	**59.4**	**NA**
	Follow-up	No detection Blood (2 sites)	7.1	80.4	155	0.59	17.2	57.6	NA
2/♂	**Admission**	*B. Pseudomallei* *Blood (2 sites)*	**12**	**89**	**50**	**11.53**	**30.5**	**86.8**	**27263**
	Follow-up	No detection Blood (2 sites)	4.9	57.2	25	1.28	15.1	73.6	NA
3/♂	**Admission**	*B. Pseudomallei* *Blood (2 sites)*	**11.6**	**79.3**	**113**	**2.12**	**19.6**	**72.9**	**NA**
	Follow-up	No detection Blood (2 sites)	7.9	77	185	0.642	16.5	70.1	NA
4/♂	**Admission**	*B. Pseudomallei* *Blood (2 sites) and ascites*	**27.5**	**91.4**	**120**	**55.26**	**125.1**	**302.9**	**2857**
	Follow-up	No detection Blood (2 sites) and ascites	4.4	74.2	59	17.93	37.7	93.4	NA
5/♀	**Admission**	*B. Pseudomallei* *Blood (2 sites), Pus*	**2.5**	**81.8**	**11**	**14.73**	**16.4**	**77.4**	**NA**
	**Deceased**	-	-	-	-	-	-	-	-
6/♀	**Admission**	*B. Pseudomallei* *Blood (2 sites)*	**5.9**	**90**	**18**	**13.42**	**40.2**	**133.51**	**2846**
	**Deceased**	-	-	-	-	-	-	-	-
7/♂	**Admission**	*B. Pseudomallei* *Blood (2 sites), sputum*	**2.9**	**66.9**	**59**	**69.35**	**48.5**	**147.5**	**7513**
	Follow-up	No detection Blood (2 sites), sputum	6.4	73.5	620	0.596	33.9	69.3	NA
8/♂	**Admission**	*B. Pseudomallei* *Blood (2 sites)*	**11.3**	**85.1**	**102**	**3.02**	**84.5**	**86.6**	**NA**
	Follow-up	No detection Blood (2 sites)	9.2	72.9	188	0.29	55.3	83.8	NA
9/♂	**Admission**	*B. Pseudomallei* *Blood (2 sites)*	**18.5**	**94.1**	**42**	**70.86**	**104.5**	**205.6**	**NA**
	Follow-up	No detection Blood (2 sites)	5.7	70.4	249	0.25	61.2	104.2	NA

Thrombocytopenia (platelet count < 150 G/L) was documented in five patients, including profound thrombocytopenia in Cases 5 and 6 (11 and 18 G/L, respectively), both of whom subsequently died. Platelet counts below 50 G/L were observed in four patients (Cases 2, 5, 6, and 9).

PCT levels were markedly elevated in all patients at admission, supporting the diagnosis of severe bacterial infection. Six patients had PCT levels exceeding 10 ng/mL, with the highest values observed in Cases 7 and 9 (69.35 and 70.86 ng/mL, respectively). Both patients required intensive care and advanced supportive management during the acute phase of illness.

Biochemical parameters also indicated multiorgan involvement. Hyperbilirubinemia was observed in six patients (Cases 2, 4, 6, 7, 8, and 9), with markedly elevated levels (>80 µmol/L) in Cases 4, 8, and 9, suggesting significant hepatic dysfunction. Serum creatinine concentrations were elevated in four patients (Cases 4, 6, 7, 9), reaching 302.9 µmol/L in Case 4 and 205.6 µmol/L in Case 9, consistent with acute kidney injury. D-dimer levels were elevated in all four patients in whom it was measured (Cases 2, 4, 6 and 7), supporting the presence of abnormalities associated with severe systemic infection.

**Fig. 3:**
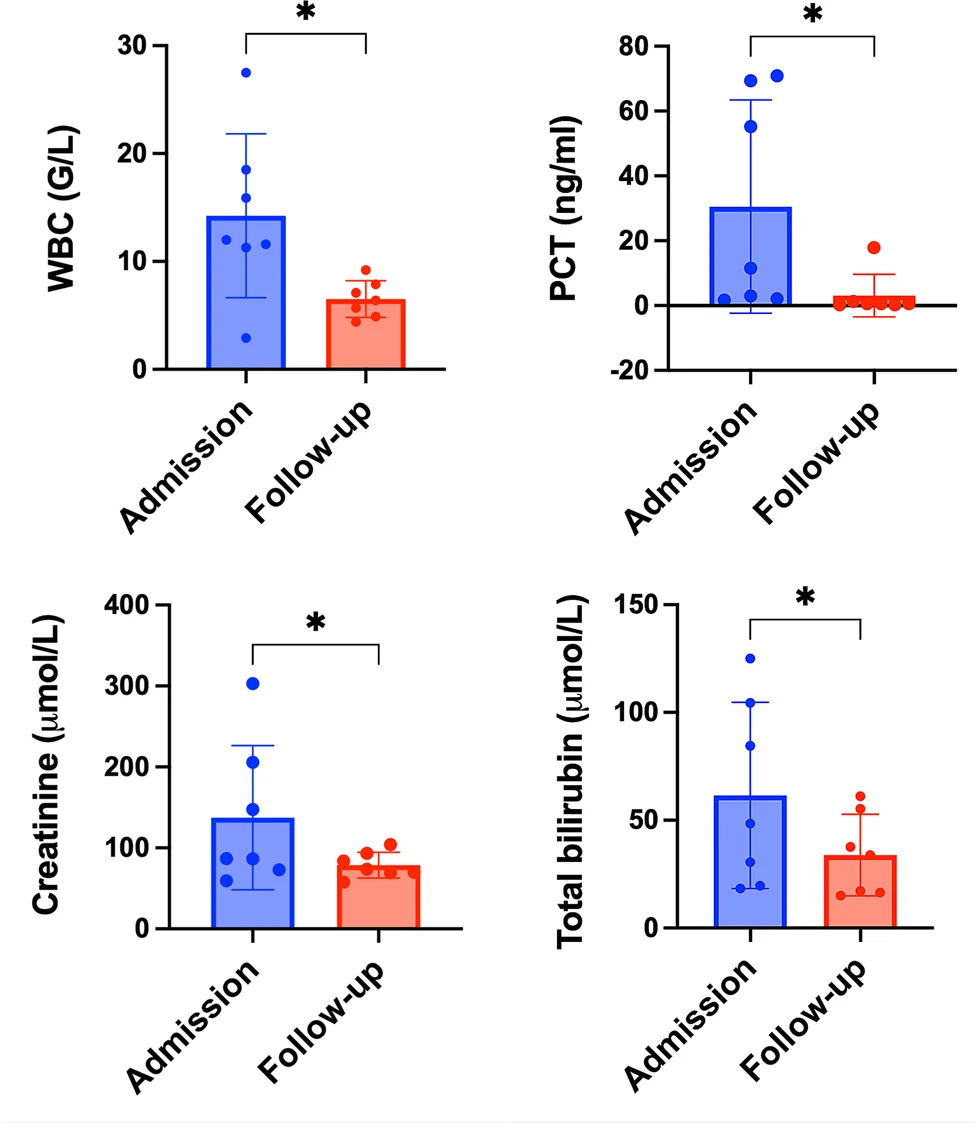
Changes in laboratory parameters between admission and the eradication phase in survivors with melioidosis. Paired column charts compare laboratory parameters at hospital admission and during the eradication phase in seven surviving patients. Shown parameters include (**A**) white blood cell count (WBC, G/L), (**B**) PCT, (ng/mL), (**C**) Serum creatinine (µmol/L), and (**D**) Total bilirubin (µmol/L). Statistical comparisons were performed using the two-tailed wilcoxon signed-rank test. Statistical significance is indicated as follows: *p < 0.05

Among the seven surviving patients who completed intensive-phase therapy and were evaluated at transition to eradication therapy, most laboratory abnormalities improved substantially during the eradication phase. Reductions were observed in inflammatory markers (WBC count and PCT), while renal and hepatic function parameters generally returned toward baseline. Follow-up blood cultures obtained during or at completion of intensive-phase therapy were negative for *B. pseudomallei* in all surviving patients, suggesting microbiological clearance (Table 3 and Fig. 3).

### Microbiological findings

**Fig. 4:**
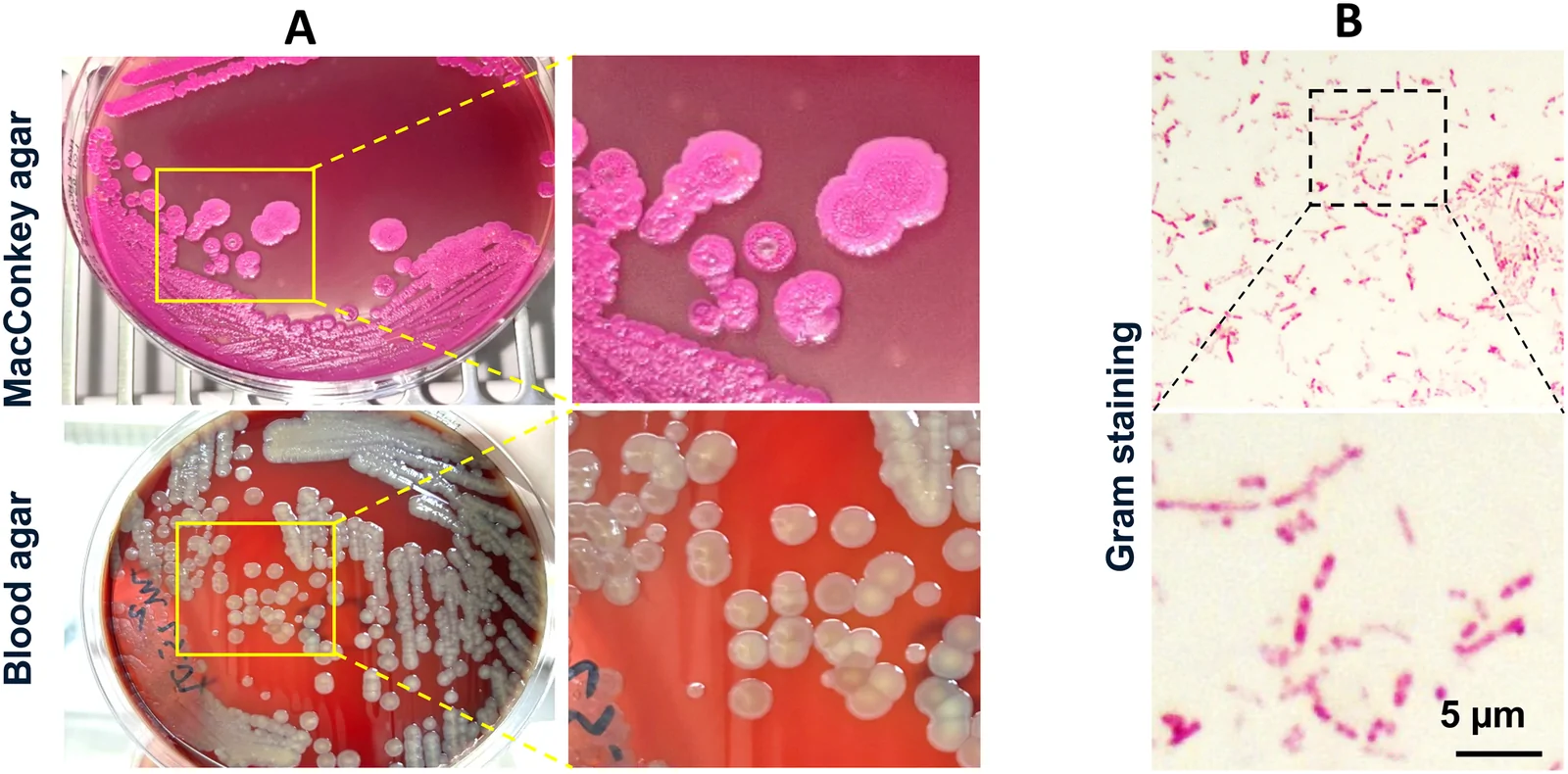
Colony morphology and Gram staining of *B. pseudomallei* isolated from clinical specimens. (**A**) Representative colony morphology of B. pseudomallei grown on MacConkey agar (upper left) and blood agar (lower left) after 48 hours of incubation. Colonies appear non–lactose-fermenting with a dry, wrinkled surface. (**B**) Gram-stained microscopic images showing small Gram-negative bacilli with characteristic bipolar (“safety-pin”) staining. Scale bar = 5 µm

Microbiological analysis of clinical specimens consistently identified *B. pseudomalle*i in all nine cases. On MacConkey agar, isolates produced non–lactose-fermenting colonies that appeared pale or slightly pink and developed a characteristic wrinkled morphology after prolonged incubation. On blood agar, colonies were creamy white to gray, non-hemolytic, slightly raised, and often exhibited a dry or wrinkled surface. Gram staining revealed small Gram-negative bacilli with bipolar staining, producing a “safety-pin” appearance, a well-recognized microscopic feature of *B. pseudomallei*. These phenotypic characteristics, in combination with culture positivity and automated identification, supported the diagnosis of melioidosis in this case series (Fig. 4). Initial identification was performed using the VITEK 2 Compact system and was subsequently confirmed by molecular testing.

### Molecular confirmation by TTS1 qPCR

**Fig. 5:**
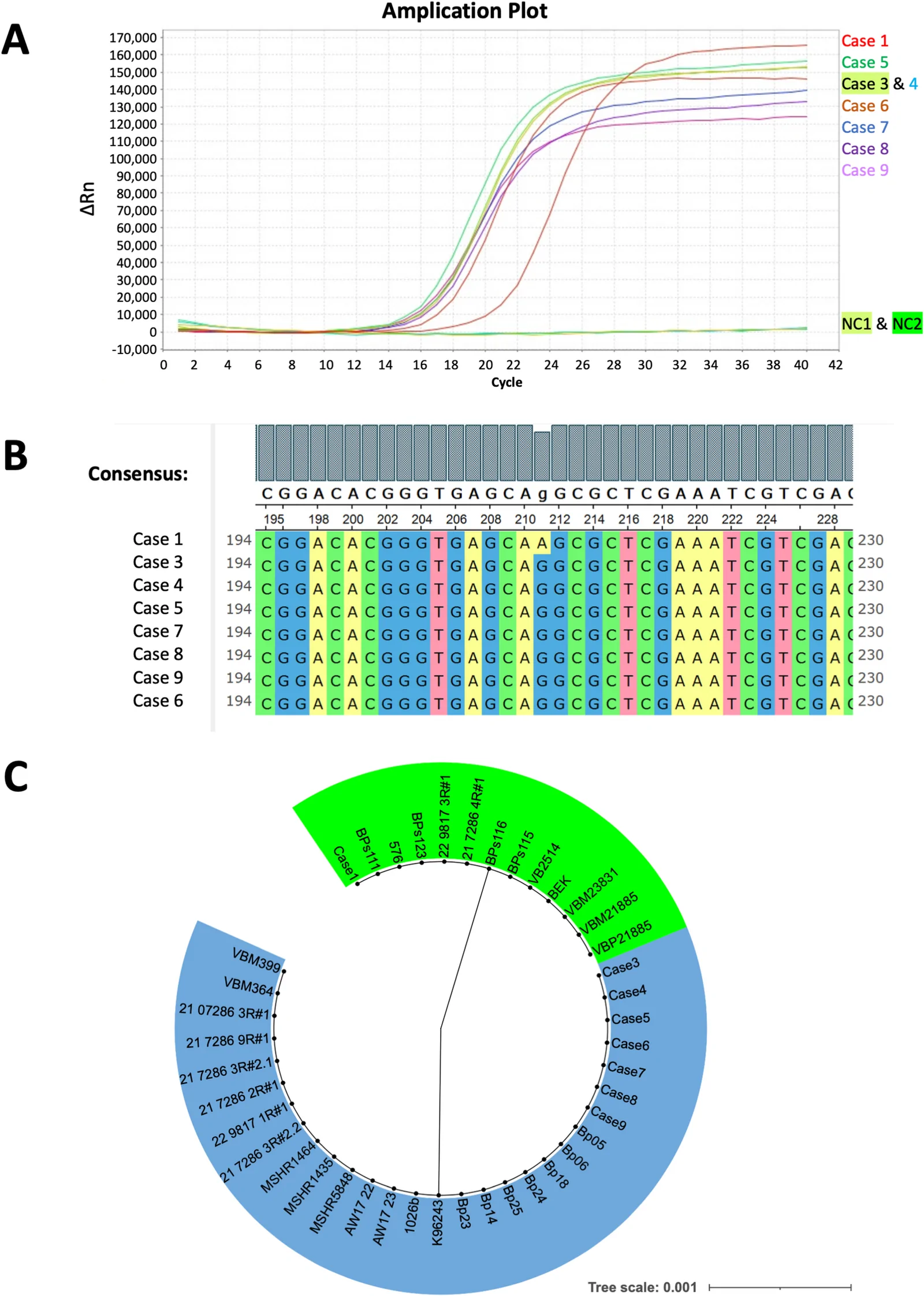
Molecular confirmation and phylogenetic analysis of *B. pseudomallei* clinical isolates. (**A**) TTS1-targeted qPCR amplification plots confirming the presence of *B. pseudomallei* DNA in eight available clinical isolates. (**B**) Alignment of the 408-bp recA gene fragment showing a single synonymous nucleotide substitution in Case 1 compared with the remaining isolates. (**C**) Maximum-likelihood phylogenetic tree illustrating the relationship between study isolates and selected reference strains from Southeast Asia and other endemic regions

To confirm species identification, qPCR targeting the type III secretion system 1 (TTS1) gene cluster was performed on available isolates. Positive amplification was obtained for isolates from Cases 1 and 3–9, with quantification cycle (Cq) values ranging from 14 to 20. No amplification was observed in the negative controls, including macrolide-resistant *Mycoplasma pneumoniae* genomic DNA and a no-template control (Fig. 5A). The isolate from Case 2 was unavailable for molecular analysis because the stored specimen could not be recovered. These findings confirmed the presence of *B. pseudomallei* in all tested isolates.

### recA sequence analysis and phylogenetic characterization

To further characterize the confirmed isolates, Sanger sequencing of the *recA* gene was performed on all TTS1-positive samples. Following sequence quality trimming, a 408-bp region was included in the comparative analysis. Sequences from Cases 3–9 were identical across the analyzed region. In contrast, the isolate from Case 1 contained a single nucleotide polymorphism (G→A) at position 211 of the analyzed fragment. This substitution corresponded to a synonymous codon change (CAG→CAA) within the coding sequence and did not alter the encoded amino acid (glutamine) (Fig. 5B).

Phylogenetic analysis based on the *recA* sequence demonstrated that the study isolates clustered with reference *B. pseudomallei* strains reported from multiple endemic regions in Asia and Oceania (Fig. 5Cand Supplementary Table S1). The Case 1 isolate grouped with sequences reported from India, Sri Lanka, Thailand, Pakistan, and New Caledonia, whereas the identical isolates from Cases 3–9 clustered with a broader group of reference strains, including environmental isolates previously recovered from Nghe An Province, Vietnam [17]. These findings suggest limited genetic variation among the analyzed *B. pseudomallei* isolates and provide the first molecular characterization of clinical melioidosis isolates from Phu Quoc Island, Vietnam.

### Treatment outcome

Empirical antimicrobial therapy at admission varied according to the initial clinical diagnosis and disease severity. Two patients (Cases 1 and 3) initially received ceftriaxone, whereas the remaining patients were treated with meropenem-based regimens. In patients presenting with severe sepsis, septic shock, or multiorgan dysfunction, combination therapy with additional agents such as amikacin, levofloxacin, or vancomycin was administered at the discretion of the treating physicians. (Table 2).

Following blood culture positivity and laboratory identification of *B. pseudomallei*, antimicrobial therapy was adjusted in accordance with institutional practice and established treatment recommendations. Meropenem was subsequently administered as the definitive intensive-phase therapy in all culture-confirmed cases, either alone or in combination with other antimicrobial agents depending on clinical severity [18].

Clinical outcomes varied according to disease severity. Seven patients required ICU or HDU admission, five underwent invasive mechanical ventilation, four required vasopressor support, and one received CVVHDF. Two patients (Cases 5 and 6) developed rapidly progressive pneumonia complicated by ARDS, septic shock, and multiple organ dysfunction syndrome, and died despite intensive antimicrobial and supportive treatment. Among the seven survivors, three achieved complete recovery at Vinmec Phu Quoc Hospital, while four were transferred to tertiary referral centers on the mainland for continuation of care during the eradication phase. No relapse was documented during follow-up (Table 2).

### Antimicrobial susceptibility

Antimicrobial susceptibility testing was retrospectively performed on eight available isolates using the broth microdilution method and interpreted according to CLSI M45 criteria. All tested isolates were susceptible to amoxicillin/clavulanic acid, ceftazidime, imipenem, and trimethoprim/sulfamethoxazole. MIC values were uniformly low across isolates, with amoxicillin/clavulanic acid ≤ 4/2 μg/mL, ceftazidime 2–4 μg/mL, imipenem ≤ 0.5 μg/mL, and trimethoprim/sulfamethoxazole ≤ 1/19 μg/mL (Table 4). No acquired resistance to first-line anti-melioidosis agents was identified among the available isolates.

**Table 4:** Summary table of antimicrobial susceptibility testing

Case	MIC (μg/mL)			
	Amoxicillin / Clavulanic acid	Ceftazidime	Imipenem	Trimethoprim / Sulfamethoxazole
1	≤4/2 (S)	2 (S)	≤0.5 (S)	≤1/19 (S)
3	≤4/2 (S)	2 (S)	≤0.5 (S)	≤1/19 (S)
4	≤4/2 (S)	2 (S)	≤0.5 (S)	≤1/19 (S)
5	≤4/2 (S)	2 (S)	≤0.5 (S)	≤1/19 (S)
6	≤4/2 (S)	2 (S)	≤0.5 (S)	≤1/19 (S)
7	≤4/2 (S)	2 (S)	≤0.5 (S)	≤1/19 (S)
8	≤4/2 (S)	2 (S)	≤0.5 (S)	≤1/19 (S)
9	≤4/2 (S)	4 (S)	≤0.5 (S)	≤1/19 (S)

S = Susceptible

## Discussion

Evidence from Vietnam over the past three decades suggests that *B. pseudomallei* is more widespread than the clinical literature alone would imply. The earliest systematic work, conducted between 1992 and 1998 in southern Vietnam, reported that *B. pseudomallei* accounted for only 0.25% of positive blood cultures, yet environmental sampling found the bacterium in 18% of rice field soil samples, with the virulent L-arabinose–negative biotype concentrated near the homes of affected patients [19]. This large discrepancy between environmental prevalence and confirmed clinical cases highlights the probability of substantial underdiagnosis—likely driven by low clinical suspicion and limited microbiological capacity in the period studied.

During the study period, Vinmec Phu Quoc International Hospital was the only healthcare facility on Phu Quoc Island capable of definitive microbiological identification of *Burkholderia pseudomallei* and routine antimicrobial susceptibility testing. Other healthcare facilities on the island lacked the laboratory capacity to reliably identify this pathogen. The identification of nine culture-confirmed cases at a single center therefore likely represents only a fraction of the true disease burden, supporting the interpretation that melioidosis is under-recognized rather than absent in this island setting.

In line with historical data from northern Vietnam, our findings reiterate that melioidosis in this region frequently presents as severe community-acquired infection associated with bacteremia, pneumonia, and sepsis, particularly among patients with underlying comorbidities [20]. Pneumonia was the predominant clinical manifestation in our cohort, occurring in seven of nine patients (77.8%). Clinical presentations ranged from localized infection to septic shock with multiorgan dysfunction, highlighting the well-recognized heterogeneity of melioidosis and its ability to mimic a wide range of infectious diseases [20]. Similar to previous Vietnamese studies, clinical manifestations in our series were often indistinguishable from other causes of severe pneumonia or sepsis, emphasizing the importance of microbiological confirmation in endemic settings [20, 21].

Our experience mirrors findings from North-Central Vietnam, where simple diagnostic algorithms combined with clinician awareness led to notable case detection and revealed high bacteremia and mortality rates (~50%) in a single health district [21]. These observations underscore the pivotal role of microbiological diagnostic capacity - particularly blood culture and accurate organism identification - in translating environmental exposure into recognized clinical disease. In resource-limited or geographically isolated settings such as Phu Quoc Island, limited access to advanced diagnostic tools may contribute to delayed diagnosis and adverse outcomes.

Although culture remains the diagnostic gold standard for melioidosis, molecular techniques may provide additional epidemiological insights. In the present study, TTS1 qPCR confirmed the identity of all tested isolates, while recA sequencing demonstrated the presence of at least two closely related sequence variants circulating on Phu Quoc Island. Most isolates shared an identical recA sequence, whereas one isolate differed by a single synonymous nucleotide substitution. Phylogenetic analysis further demonstrated close relatedness to previously reported clinical and environmental isolates from Vietnam and neighboring endemic countries. Although recA sequencing provides lower discriminatory power than multilocus sequence typing or whole-genome sequencing, these findings support the existence of genetic diversity among *B. pseudomallei* strains circulating locally and confirm the taxonomic identity of the study isolates [5, 22].

The epidemiological findings of this study further support the existence of an environmental reservoir of *B. pseudomallei* on Phu Quoc Island. All patients were permanent residents of the island, and in more than half of the cases no specific exposure source could be identified. Nevertheless, the occurrence of multiple culture-confirmed cases over several years, together with the known environmental distribution of *B. pseudomallei* in Vietnam, strongly suggests ongoing local exposure rather than sporadic importation [20, 21]. While environmental sampling was not performed, these observations support the hypothesis that Phu Quoc Island represents an endemic setting for melioidosis.

In our cohort, diabetes mellitus was the most prevalent comorbidity, affecting seven of nine patients (77.8%), followed by cirrhosis, chronic liver disease, and hazardous alcohol use. This pattern closely mirrors findings from referral hospitals in Vietnam and other endemic regions, where diabetes consistently emerges as the strongest risk factor for melioidosis [15]. These host conditions are well recognized to impair innate immune responses - particularly neutrophil function and cell-mediated immunity - thereby increasing susceptibility to invasive disease and severe clinical manifestations. The predominance of such comorbidities among patients with severe or septicemic presentations in our cohort further supports the central role of host susceptibility, rather than exposure alone, in determining disease severity and clinical outcome [23–25].

Several patients presented with potential portals of entry involving the upper respiratory tract or breaches in skin integrity. Cases 5, 6, and 9 developed systemic illness following preceding upper respiratory tract infections, including purulent tonsillitis, rhinitis with fever, and acute purulent pharyngitis, respectively. Case 8 developed severe cellulitis at the site of repeated intramuscular injections in the thigh. These observations suggest that mucosal inflammation or local tissue injury may facilitate bacterial invasion in endemic settings. However, such factors likely represent portals of entry rather than the primary environmental source of infection, which remains difficult to establish retrospectively.

Neurological manifestations were observed in two patients. Case 3 presented with meningoencephalitis associated with diabetic ketoacidosis, whereas Case 2 developed an intracerebral hemorrhage after initial clinical improvement. Neuroimaging demonstrated a deep right hemispheric intracerebral hemorrhage with intraventricular extension and surrounding cerebral edema but no evidence of an underlying structural cerebrovascular lesion. Although severe sepsis-associated coagulopathy may have contributed to the hemorrhagic event, a direct causal relationship between melioidosis and intracerebral hemorrhage cannot be established from the available data. Nevertheless, this case highlights the potential complexity of neurological complications occurring during severe systemic infection.

Notably, both fatal cases (Cases 5 and 6) demonstrated rapidly progressive pulmonary disease. Initial chest imaging showed relatively limited interstitial and alveolar abnormalities, but extensive bilateral pulmonary infiltrates developed within days, progressing to ARDS, septic shock, and multiorgan dysfunction syndrome. Case 5 was a previously healthy young woman without recognized predisposing conditions, whereas Case 6 was an elderly patient with poorly controlled diabetes mellitus. Despite differences in baseline risk factors, both experienced fulminant pulmonary melioidosis with fatal outcomes. Similar rapidly progressive pulmonary presentations have been reported in other endemic regions and underscore the importance of early recognition and prompt initiation of effective antimicrobial therapy [7, 26].

The occurrence of fatal disease in a previously healthy patient is particularly noteworthy. Although severe melioidosis is classically associated with diabetes, chronic kidney disease, liver disease, or hazardous alcohol use, severe infection may also occur in individuals without recognized risk factors [7, 26, 27]. A large cohort study from northeastern Thailand demonstrated that patients without known risk factors did not experience lower mortality than those with established comorbidities [7]. Accordingly, the absence of traditional risk factors should not lower clinical suspicion in patients presenting with severe pneumonia or sepsis in endemic areas.

All tested isolates in our study were susceptible to amoxicillin/clavulanic acid, ceftazidime, imipenem, and trimethoprim/sulfamethoxazole when interpreted according to CLSI M45 broth microdilution criteria. These findings are consistent with reports from Vietnam and other endemic regions, where primary resistance among *B. pseudomallei* remains uncommon [28–30]. The uniformly favorable susceptibility profile observed in our series supports current treatment recommendations using ceftazidime or carbapenems during the intensive phase followed by prolonged oral eradication therapy [18, 28–30].

Given the high proportion of patients presenting with severe disease, empirical antimicrobial therapy frequently included meropenem-based regimens before microbiological confirmation. Following culture identification of *B. pseudomallei,* treatment was adjusted according to established melioidosis management guidelines. These observations reinforce the importance of early effective therapy, as delayed initiation of active antimicrobial treatment remains one of the strongest predictors of mortality in melioidosis [18].

Melioidosis remains a challenging infectious disease with variable clinical presentations and substantial mortality. Our findings suggest that in island settings such as Phu Quoc—where environmental exposure likely exists but diagnostic awareness remains limited—clinicians should maintain a high index of suspicion for melioidosis in patients presenting with pneumonia, sepsis, or septic shock, particularly during the rainy season and among individuals with diabetes or chronic liver disease. Routine blood cultures, accurate laboratory identification of *B. pseudomallei*, and timely initiation of appropriate antimicrobial therapy are likely to have a greater impact on patient outcomes than access to advanced molecular technologies alone [5, 27].

This study has several limitations. The retrospective design and small sample size limit the generalizability of our findings. Environmental sampling was not performed, precluding direct confirmation of local reservoirs. In addition, molecular characterization was limited to TTS1 qPCR and partial recA sequencing; higher-resolution approaches such as multilocus sequence typing or whole-genome sequencing may provide additional insights into transmission dynamics and strain diversity. Nevertheless, the combination of detailed clinical, microbiological, radiological, antimicrobial susceptibility, and molecular data provides valuable information regarding melioidosis in an under-recognized island setting.

Taken together, our findings support three key messages. First, melioidosis is likely endemic but under-recognized on Phu Quoc Island. Second, host susceptibility—particularly diabetes mellitus and chronic liver disease—appears to play a major role in determining disease severity, although fulminant disease may also occur in previously healthy individuals. Third, strengthening conventional microbiological capacity, including blood culture, organism identification, and susceptibility testing, remains the most practical strategy for improving diagnosis and outcomes in resource-limited or geographically isolated settings.

## Conclusion

Melioidosis should be recognized as an endemic yet under-recognized infectious disease on Phu Quoc Island and should be considered in the differential diagnosis of severe pneumonia, sepsis, and rapidly progressive respiratory failure, particularly during the rainy season. Although diabetes mellitus and chronic liver disease were common predisposing factors in our cohort, fulminant disease also occurred in a previously healthy individual, indicating that the absence of traditional risk factors does not exclude severe melioidosis.

The heterogeneous clinical and radiological manifestations observed in this series highlight the need for clinicians to maintain a high index of suspicion, especially when patients present with pneumonia and sepsis that progress rapidly despite standard empirical therapy. Early recognition, timely blood culture, accurate microbiological identification of *Burkholderia pseudomallei*, and prompt initiation of effective intensive-phase antimicrobial therapy remain critical for improving outcomes.

All tested isolates were susceptible to recommended first-line antimicrobial agents, supporting current treatment guidelines. In endemic island settings such as Phu Quoc, strengthening conventional microbiological capacity and increasing clinician awareness are likely to have a greater immediate impact on patient survival than access to advanced molecular diagnostics. Greater recognition of melioidosis in Vietnam’s island and coastal regions may reduce diagnostic delays and improve outcomes for this potentially fatal but treatable disease.

## Electronic supplementary material

Below is the link to the electronic supplementary material.

Supplementary Material 1

## Data Availability

All data is available in the article and tables. The datasets used and/or analyzed in the current study are available from the contact author upon reasonable request.
