# Supplementary material for: Melioidosis on Phu Quoc Island, Vietnam: a retrospective case series from a remote tropical setting
Source: BMC Infect Dis. 2026 Aug 13;26:1978. doi: 10.1186/s12879-026-14150-4 (PMC13753210; doi:10.1186/s12879-026-14150-4)
Supplement: Supplementary file 1 [file 12879_2026_14150_MOESM1_ESM.docx]

**Supplement Data**

Supplementary Table S1. Reference *Burkholderia pseudomallei* strains included in the phylogenetic analysis.

| **Strain** | **GenBank ID** | **Country** | **Year** |
| --- | --- | --- | --- |
| BPs111 | [CP036454.1](https://www.ncbi.nlm.nih.gov/nucleotide/CP036454.1?report=genbank&log$=nucltop&blast_rank=1&RID=28VE63D8014) | Sir Lanka | 2015 |
| 576 | [CP008777.1](https://www.ncbi.nlm.nih.gov/nucleotide/CP008777.1?report=genbank&log$=nucltop&blast_rank=2&RID=28VE63D8014) | Thailand | N/A |
| BPs123 | [CP037970.1](https://www.ncbi.nlm.nih.gov/nucleotide/CP037970.1?report=genbank&log$=nucltop&blast_rank=3&RID=28VE63D8014) | Sri Lanka | 2015 |
| 22-9817_3 R#1 | [OZ241391.1](https://www.ncbi.nlm.nih.gov/nucleotide/OZ241391.1?report=genbank&log$=nucltop&blast_rank=4&RID=28VE63D8014) | New Caledonia | 2022 |
| 21-7286_4 R#1 | [OZ241825.1](https://www.ncbi.nlm.nih.gov/nucleotide/OZ241825.1?report=genbank&log$=nucltop&blast_rank=5&RID=28VE63D8014) | New Caledonia | 2021 |
| BPs116 | [CP037760.1](https://www.ncbi.nlm.nih.gov/nucleotide/CP037760.1?report=genbank&log$=nucltop&blast_rank=6&RID=28VE63D8014) | Sri Lanka | 2015 |
| BPs115 | [CP037758.1](https://www.ncbi.nlm.nih.gov/nucleotide/CP037758.1?report=genbank&log$=nucltop&blast_rank=7&RID=28VE63D8014) | Sri Lanka | 2015 |
| VB2514 | [CP040552.1](https://www.ncbi.nlm.nih.gov/nucleotide/CP040552.1?report=genbank&log$=nucltop&blast_rank=8&RID=28VE63D8014) | India | 2019 |
| BEK | [CP008755.1](https://www.ncbi.nlm.nih.gov/nucleotide/CP008755.1?report=genbank&log$=nucltop&blast_rank=9&RID=28VE63D8014) | Pakistan | N/A |
| VBM23831 | [CP071751.1](https://www.ncbi.nlm.nih.gov/nucleotide/CP071751.1?report=genbank&log$=nucltop&blast_rank=10&RID=28VE63D8014) | India | 2020 |
| VBM21885 | [CP071749.1](https://www.ncbi.nlm.nih.gov/nucleotide/CP071749.1?report=genbank&log$=nucltop&blast_rank=11&RID=28VE63D8014) | India | 2020 |
| VBP21885 | [CP071759.1](https://www.ncbi.nlm.nih.gov/nucleotide/CP071759.1?report=genbank&log$=nucltop&blast_rank=12&RID=28VE63D8014) | India | 2020 |
| Bp11 | CP193463.1 | Vietnam | 2017 |
| Bp16 | CP193455.1 | Vietnam | 2017 |
| Bp05 | CP193468.1 | Vietnam | 2017 |
| Bp06 | CP193466.1 | Vietnam | 2017 |
| Bp18 | CP193452.1 | Vietnam | 2017 |
| Bp24 | CP193444.1 | Vietnam | 2017 |
| Bp25 | CP193441.1 | Vietnam | 2017 |
| Bp14 | CP193459.1 | Vietnam | 2017 |
| Bp23 | CP193445.1 | Vietnam | 2017 |
| K96243 | CP009538.1 | Thailand | 1993 |
| 1026b | CP004379.1 | Thailand | 1993 |
| AW17-23 | CP073734.1 | Singapore | 2019 |
| AW17-22 | CP073737.1 | Singapore | 2019 |
| MSHR5848 | CP008909.1 | Australia | 2011 |
| MSHR1435 | CP025264.1 | Australia | 2002 |
| MSHR1464 | CP111141.1 | Australia | 2003 |
| 21-7286_3 R#2.2 | OZ241178.1 | New Caledonia | 2021 |
| 22-9817_1 R#1 | OZ242944.1 | New Caledonia | 2022 |
| 21-7286_2 R#1 | OZ242940.1 | New Caledonia | 2021 |
| 21-7286_3 R#2.1 | OZ241174.1 | New Caledonia | 2021 |
| 21-7286_9 R#1 | OZ241213.1 | New Caledonia | 2021 |
| 21-07286_3 R#1 | OZ241168.1 | New Caledonia | 2021 |
| VBM364 | CP071525.2 | India | 2020 |
| VBM399 | CP071526.2 | India | 2020 |
